# The genome sequence of the Lesser Skullcap, *Scutellaria minor *Huds., 1762 (Lamiaceae)

**DOI:** 10.12688/wellcomeopenres.21164.1

**Published:** 2024-03-20

**Authors:** Sahr Mian, Maarten J. M. Christenhusz, Ilia J Leitch, Royal Botanic Gardens Kew Genome Acquisition Lab, Plant Genome Sizing collective, Darwin Tree of Life Barcoding collective, Wellcome Sanger Institute Tree of Life Management, Samples and Laboratory team, Wellcome Sanger Institute Scientific Operations: Sequencing Operations, Wellcome Sanger Institute Tree of Life Core Informatics team, Tree of Life Core Informatics collective, Darwin Tree of Life Consortium

**Affiliations:** 1 Royal Botanic Gardens Kew, Richmond, England, UK

**Keywords:** Scutellaria minor, Lesser Skullcap, genome sequence, chromosomal, Lamiales

## Abstract

We present a genome assembly from an individual *Scutellaria minor* (Tracheophyta; Magnoliopsida; Lamiales; Lamiaceae). The genome sequence is 341.8 megabases in span. Most of the assembly is scaffolded into 14 chromosomal pseudomolecules. The mitochondrial and plastid genome assemblies have lengths of 376.64 kilobases and 152.59 kilobases in length, respectively.

## Species taxonomy

Eukaryota; Viridiplantae; Streptophyta; Streptophytina; Embryophyta; Tracheophyta; Euphyllophyta; Spermatophyta; Magnoliopsida; Mesangiospermae; eudicotyledons; Gunneridae; Pentapetalae; asterids; lamiids; Lamiales; Lamiaceae; Scutellarioideae; *Scutellaria*; *Scutellaria minor* Huds., 1762 (NCBI:txid1053395).

## Background

*Scutellaria minor* is a perennial herb usually found in wet heaths, bogs, marshes and generally acidic soils. Belonging to the mint family (Lamiaceae), the plant grows up to 25 cm tall with small pinkish flowers and is distributed through the southern temperate regions of Europe ( Stace *et al.*, 2019). Found mainly in the southern regions of Britain and Ireland, *S*. *minor* populations have since declined, particularly in the Midlands, due to drainage and loss of habitat ( Stroh *et al.*, 2023).

Several species within the genus *Scutellaria* have historically been used in traditional medicinal practices to treat ailments and disease, including respiratory, cardiovascular and neurological diseases ( Grzegorczyk-Karolak *et al.*, 2016; Shen *et al.*, 2021). Other species within the genus have proven anticancer, anti-bacterial and anti-inflammatory properties ( Cheng *et al.*, 2018; de Boer *et al.*, 2005). The variety of bioactive compounds found in the leaves and roots of this genus have been studied extensively due to their potential medicinal value, with more than 295 compounds having been identified so far ( Georgieva *et al.*, 2019; Shang *et al.*, 2010). The two main groups of bioactive compounds found in *Scutellaria* are flavonoids and diterpenes, both of which are found in high concentrations ( Georgieva *et al.*, 2019; Shang *et al.*, 2010). Flavonoids and their derivatives have been confirmed to have anti-tumour, anti-mutagenic, neuroprotective and hepatoprotective properties ( Shang *et al.*, 2010). While the diterpenoids isolated from *Scutellaria* have been of interest due to their biological pest control potential as anti-feedant and anti-fungal agents to prevent damage to economically important crops ( Cole *et al.*, 1990).

Cytologically, studies have reported this species to have a chromosome count of either 2n = 28 (e.g. in material from Sweden) or 2n = c. 32 (e.g. material from UK) ( Henniges *et al.*, 2022; Morton, 1973; Ranjbar & Mahmoudi, 2013). In both cases the authors consider this species to be a tetraploid given that other species in the genus have been reported to have 2 *n* = 14 or 2 *n* = 16. Nevertheless, whether the species is an auto- or allo-polyploid is currently unclear.

The whole genome assembly of *S. minor* is expected to elucidate further properties and uses of the bioactive chemicals present within this diverse genus.

## Genome sequence report

**Figure 1.**
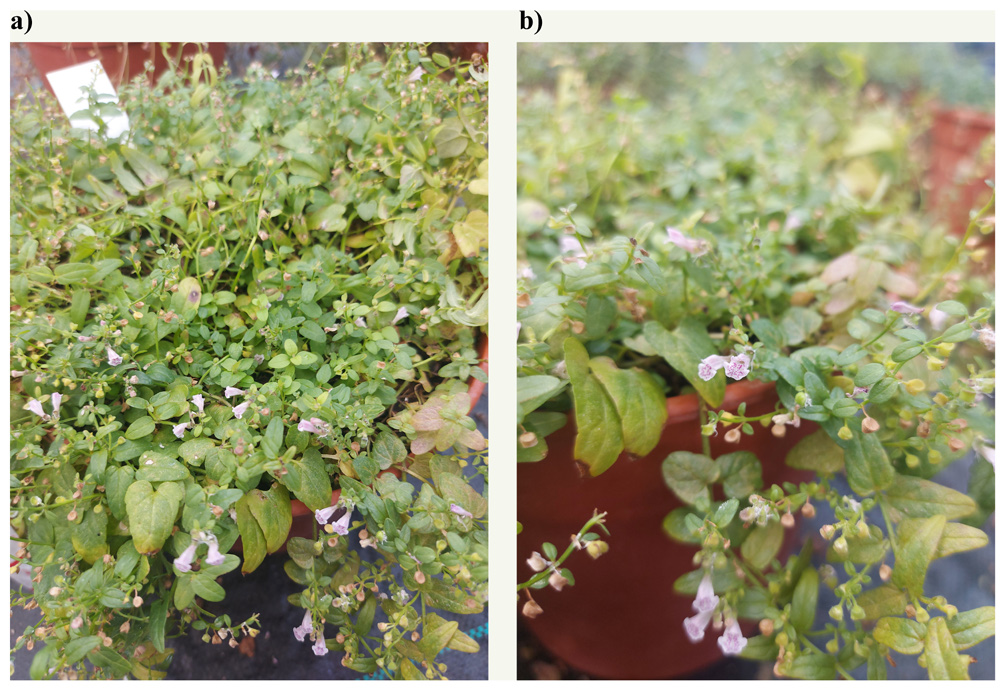
Photograph of the *Scutellaria minor* (daScuMino1) specimen used for genome sequencing.

The genome was sequenced from one specimen of *Scutellaria minor* ( Figure 1) collected from Jodrell Laboratory Glasshouses at the Royal Botanic Gardens Kew, where it was grown from seed from the Millennium Seed Bank (seed accession number 678632). Using flow cytometry, the genome size (1C-value) was estimated to be 0.43 pg, equivalent to 420 Mb. A total of 54-fold coverage in Pacific Biosciences single-molecule HiFi long reads was generated. Primary assembly contigs were scaffolded with chromosome conformation Hi-C data. Manual assembly curation corrected 3 missing joins or mis-joins, reducing the scaffold number by 15.38%.

**Figure 2.**
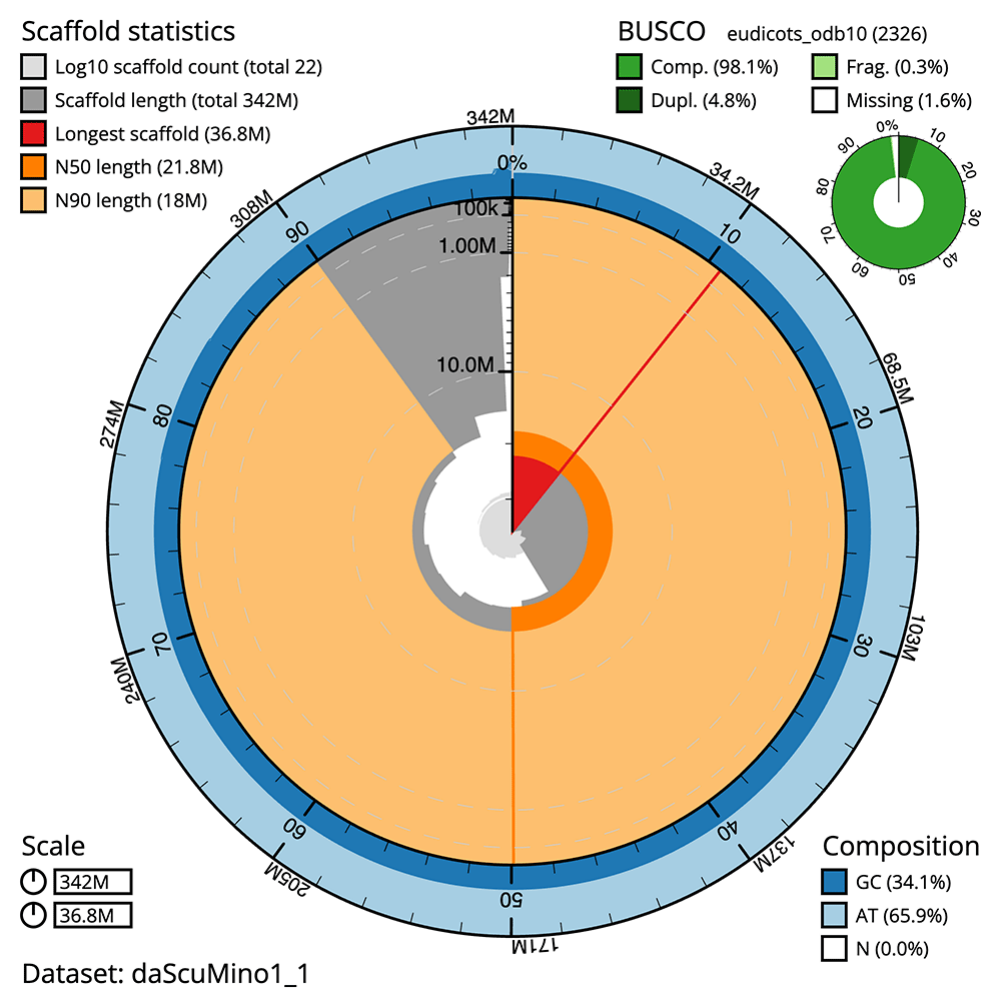
Genome assembly of *Scutellaria minor*, daScuMino1.1: metrics. The BlobToolKit Snailplot shows N50 metrics and BUSCO gene completeness. The main plot is divided into 1,000 size-ordered bins around the circumference with each bin representing 0.1% of the 342,281,700 bp assembly. The distribution of scaffold lengths is shown in dark grey with the plot radius scaled to the longest scaffold present in the assembly (36,764,200 bp, shown in red). Orange and pale-orange arcs show the N50 and N90 scaffold lengths (21,760,285 and 17,994,855 bp), respectively. The pale grey spiral shows the cumulative scaffold count on a log scale with white scale lines showing successive orders of magnitude. The blue and pale-blue area around the outside of the plot shows the distribution of GC, AT and N percentages in the same bins as the inner plot. A summary of complete, fragmented, duplicated and missing BUSCO genes in the eudicots_odb10 set is shown in the top right. An interactive version of this figure is available at https://blobtoolkit.genomehubs.org/view/daScuMino1_1/dataset/daScuMino1_1/snail.

**Figure 3.**
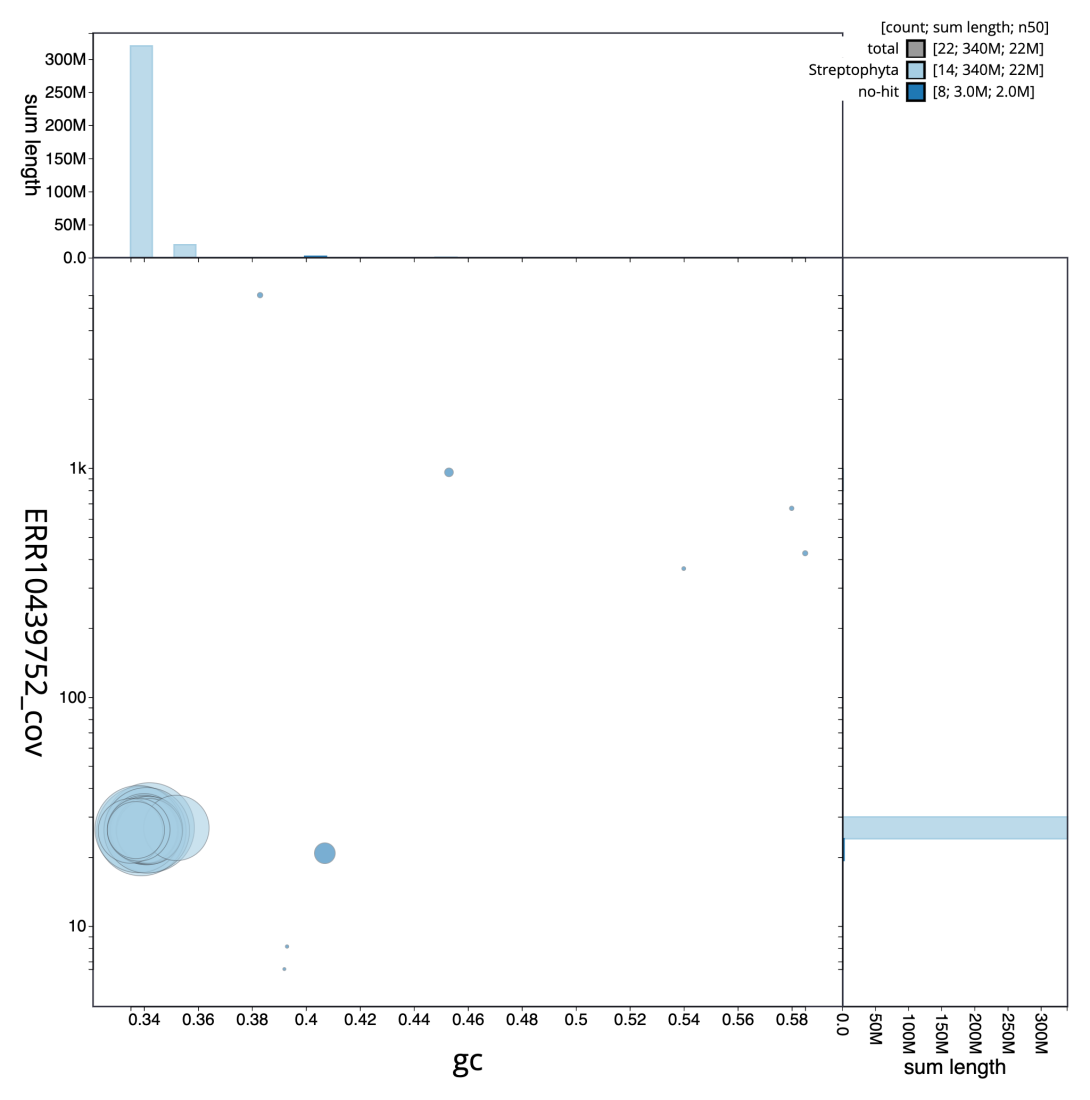
Genome assembly of *Scutellaria minor*, daScuMino1.1: BlobToolKit GC-coverage plot. Scaffolds are coloured by phylum. Circles are sized in proportion to scaffold length. Histograms show the distribution of scaffold length sum along each axis. An interactive version of this figure is available at https://blobtoolkit.genomehubs.org/view/daScuMino1_1/dataset/daScuMino1_1/blob.

**Figure 4.**
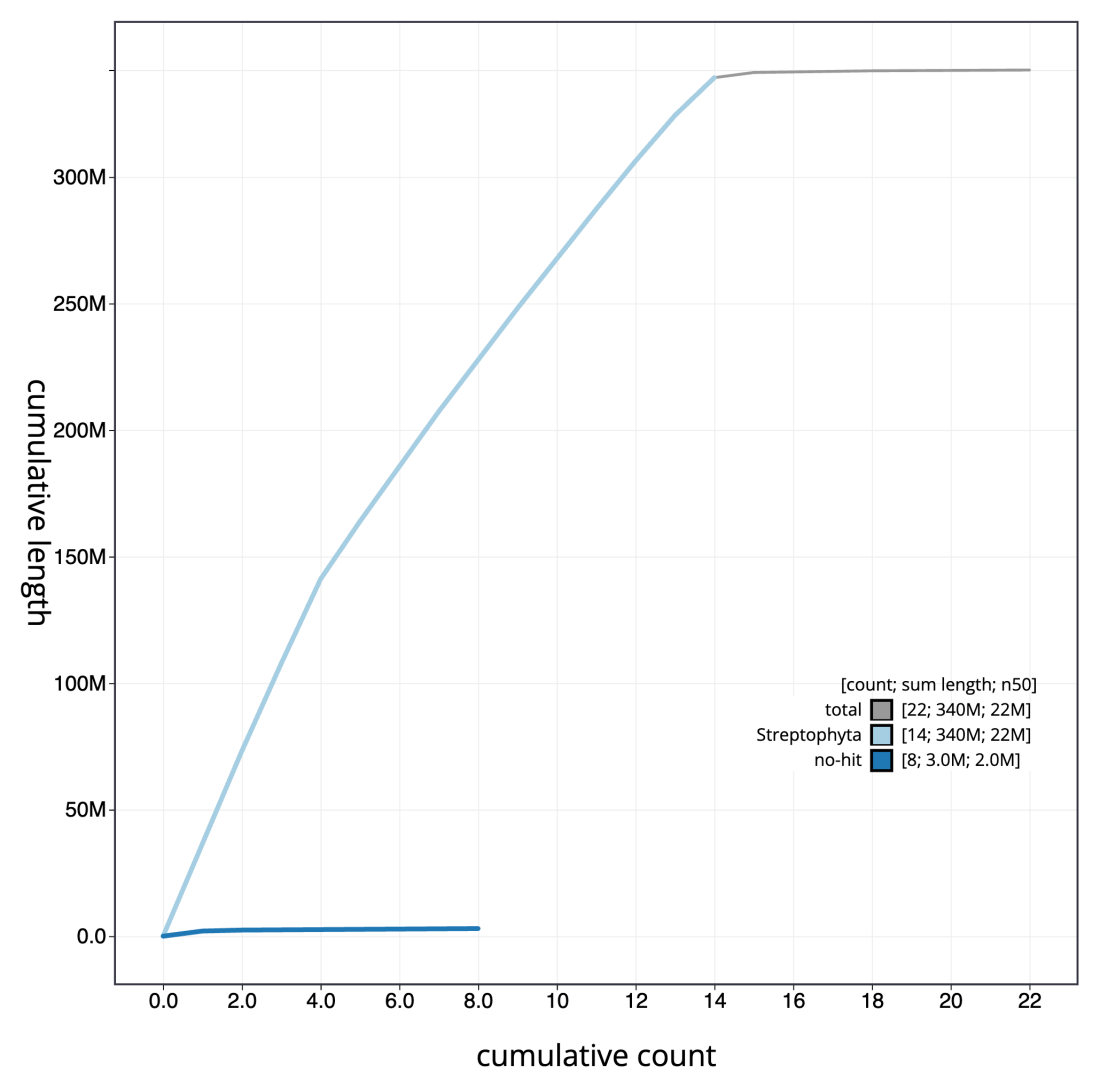
Genome assembly of *Scutellaria minor*, daScuMino1.1: BlobToolKit cumulative sequence plot. The grey line shows cumulative length for all scaffolds. Coloured lines show cumulative lengths of scaffolds assigned to each phylum using the buscogenes taxrule. An interactive version of this figure is available at https://blobtoolkit.genomehubs.org/view/daScuMino1_1/dataset/daScuMino1_1/cumulative.

**Figure 5.**
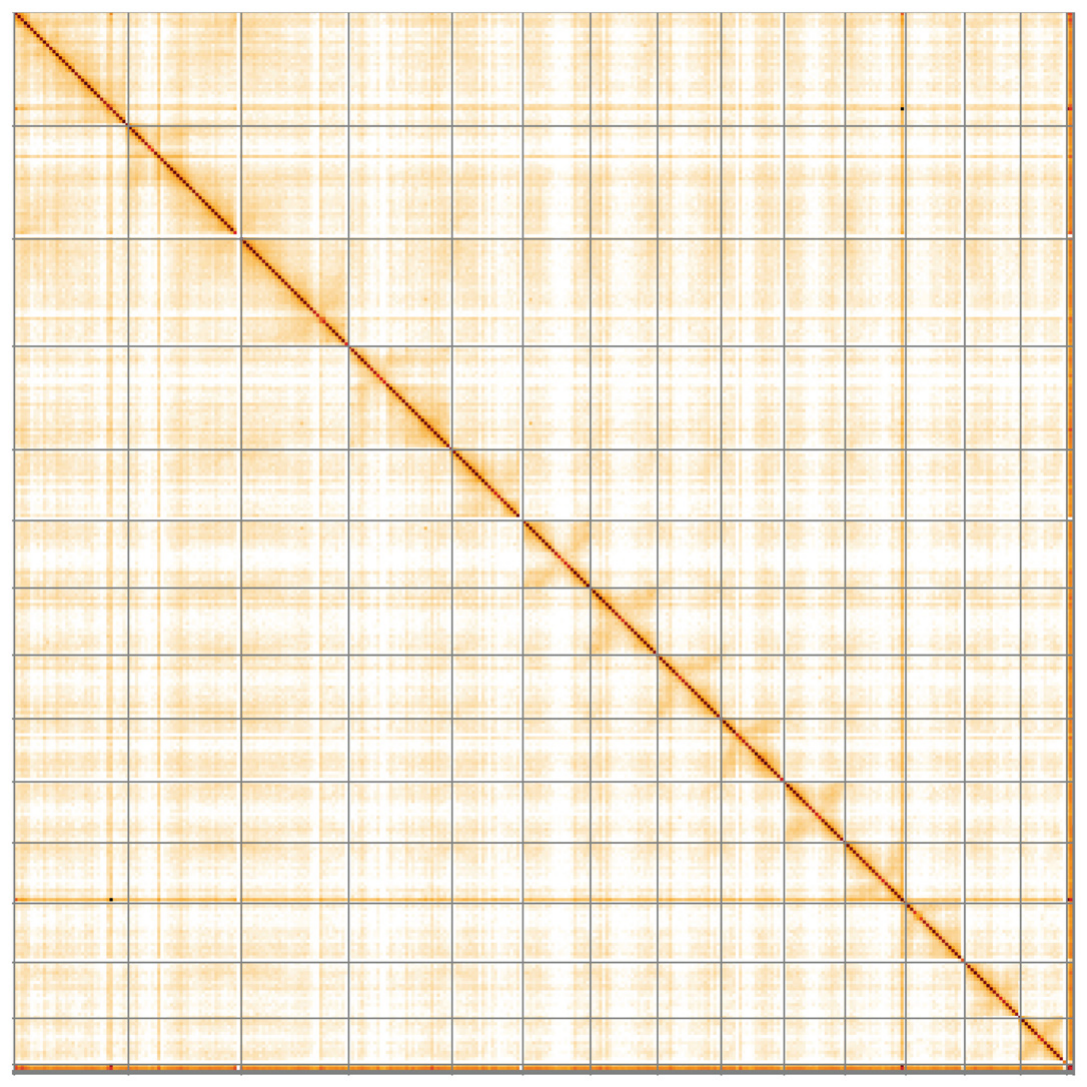
Genome assembly of *Scutellaria minor*, daScuMino1.1: Hi-C contact map of the daScuMino1.1 assembly, visualised using HiGlass. Chromosomes are shown in order of size from left to right and top to bottom. An interactive version of this figure may be viewed at https://genome-note-higlass.tol.sanger.ac.uk/l/?d=S8hyWCfwRq2qMhenu7cSyA.

The final assembly has a total length of 341.8 Mb in 20 sequence scaffolds with a scaffold N50 of 21.8 Mb ( Table 1). The snail plot in Figure 2 provides a summary of the assembly statistics, while the distribution of assembly scaffolds on GC proportion and coverage is shown in Figure 3. The cumulative assembly plot in Figure 4 shows curves for subsets of scaffolds assigned to different phyla. Most (99.12%) of the assembly sequence was assigned to 14 chromosomal-level scaffolds, indicating the individual sequenced has a chromosome count of 2n = 28. Chromosome-scale scaffolds confirmed by the Hi-C data are named in order of size ( Figure 5; Table 2). While not fully phased, the assembly deposited is of one haplotype. Contigs corresponding to the second haplotype have also been deposited. The mitochondrial and plastid genomes were also assembled and can be found as contigs within the multifasta file of the genome submission.

**Table 1.** Genome data for *Scutellaria minor*, daScuMino1.1.

Project accession data		
Assembly identifier	daScuMino1.1	
Species	*Scutellaria minor*	
Specimen	daScuMino1	
NCBI taxonomy ID	1053395	
BioProject	PRJEB57113	
BioSample ID	SAMEA7522634	
Isolate information	daScuMino1: leaf (DNA, Hi-C and RNA sequencing)	
Assembly metrics *		*Benchmark*
Consensus quality (QV)	67.8	*≥ 50*
*k*-mer completeness	100.0%	*≥ 95%*
BUSCO **	C:98.1%[S:93.3%,D:4.8%], F:0.3%,M:1.6%,n:2,326	*C ≥ 95%*
Percentage of assembly mapped to chromosomes	99.12%	*≥ 95%*
Sex chromosomes	-	*localised homologous pairs*
Organelles	Mitochondrial genome: 376.64 kb Plastid genome: 152.59 kb	*complete single alleles*
Raw data accessions		
PacificBiosciences SEQUEL II	ERR10439752	
Hi-C Illumina	ERR10446387	
PolyA RNA-Seq Illumina	ERR10446386	
Genome assembly		
Assembly accession	GCA_954870855.1	
*Accession of alternate haplotype*	GCA_954870745.1	
Span (Mb)	341.8	
Number of contigs	28	
Contig N50 length (Mb)	19.7	
Number of scaffolds	20	
Scaffold N50 length (Mb)	21.8	
Longest scaffold (Mb)	36.76	

* Assembly metric benchmarks are adapted from column VGP-2020 of “Table 1: Proposed standards and metrics for defining genome assembly quality” from ( Rhie *et al.*, 2021). ** BUSCO scores based on the eudicots_odb10 BUSCO set using version 5.3.2. C = complete [S = single copy, D = duplicated], F = fragmented, M = missing, n = number of orthologues in comparison. A full set of BUSCO scores is available at https://blobtoolkit.genomehubs.org/view/daScuMino1_1/dataset/daScuMino1_1/busco.

**Table 2.** Chromosomal pseudomolecules in the genome assembly of *Scutellaria minor*, daScuMino1.

INSDC accession	Chromosome	Length (Mb)	GC%
OX940725.1	1	36.76	34.0
OX940726.1	2	36.39	34.0
OX940727.1	3	34.62	34.0
OX940728.1	4	33.34	34.0
OX940729.1	5	22.82	34.0
OX940730.1	6	21.76	34.0
OX940731.1	7	21.63	34.0
OX940732.1	8	20.51	34.0
OX940733.1	9	20.3	34.0
OX940734.1	10	19.68	34.0
OX940735.1	11	19.52	35.0
OX940736.1	12	19.04	33.5
OX940737.1	13	17.99	34.0
OX940738.1	14	14.91	33.5
OX940739.1	MT	0.38	45.5
OX940740.1	Pltd	0.15	38.5

The estimated Quality Value (QV) of the final assembly is 67.8 with *k*-mer completeness of 100.0%, and the assembly has a BUSCO v5.3.2 completeness of 98.1% (single = 93.3%, duplicated = 4.8%), using the eudicots_odb10 reference set ( *n* = 2,326).

Metadata for specimens, barcode results, spectra estimates, sequencing runs, contaminants and pre-curation assembly statistics are given at https://links.tol.sanger.ac.uk/species/1053395.

## Methods

### Sample acquisition, genome size estimation and nucleic acid extraction

A specimen of *Scutellaria minor* (specimen ID KDTOL10114, ToLID daScuMino1) was picked by hand from the Jodrell Laboratory Glasshouses, Royal Botanic Gardens Kew, Richmond, UK (latitude 51.48, longitude –0.29) on 2020-09-10. The specimen was grown from seed from the Millennium Seed Bank. The specimen was collected by Sahr Mian (Royal Botanic Gardens, Kew) and identified by Maarten Christenhusz (Royal Botanic Gardens, Kew) and frozen at –80 °C.

The genome size was estimated by flow cytometry using the fluorochrome propidium iodide and following the ‘one-step’ method as outlined in Pellicer *et al*. (2021). The General Purpose Buffer (GPB) supplemented with 3% PVP and 0.08% (v/v) beta-mercaptoethanol was used for isolation of nuclei ( Loureiro *et al.*, 2007), and the internal calibration standard was *Solanum lycopersicum* ‘Stupiké polní rané’ with an assumed 1C-value of 968 Mb ( Doležel *et al.*, 2007).

The workflow for high molecular weight (HMW) DNA extraction at the Wellcome Sanger Institute (WSI) includes a sequence of core procedures: sample preparation; sample homogenisation, DNA extraction, fragmentation, and clean-up. In sample preparation, the daScuMino1 sample was weighed and dissected on dry ice ( Jay *et al.*, 2023). Leaf tissue was homogenised by cryogenic bead beating ( Jackson & Howard, 2023a). HMW DNA was extracted using the Automated Plant MagAttract v4 protocol ( Jackson & Howard, 2023b). HMW DNA was sheared into an average fragment size of 12–20 kb in a Megaruptor 3 system with speed setting 31 ( Bates *et al.*, 2023). Sheared DNA was purified by solid-phase reversible immobilisation ( Oatley *et al.*, 2023): in brief, the method employs a 1.8X ratio of AMPure PB beads to sample to eliminate shorter fragments and concentrate the DNA. The concentration of the sheared and purified DNA was assessed using a Nanodrop spectrophotometer and Qubit Fluorometer and Qubit dsDNA High Sensitivity Assay kit. Fragment size distribution was evaluated by running the sample on the FemtoPulse system.

RNA was extracted from leaf tissue of daScuMino1 in the Tree of Life Laboratory at the WSI using the RNA Extraction: Automated MagMax™ *mir*Vana protocol ( do Amaral *et al.*, 2023). The RNA concentration was assessed using a Nanodrop spectrophotometer and a Qubit Fluorometer using the Qubit RNA Broad-Range Assay kit. Analysis of the integrity of the RNA was done using the Agilent RNA 6000 Pico Kit and Eukaryotic Total RNA assay.

Protocols developed by the WSI Tree of Life core laboratory are publicly available on protocols.io ( Denton *et al.*, 2023).

### Sequencing

Pacific Biosciences HiFi circular consensus DNA sequencing libraries were constructed according to the manufacturers’ instructions. Poly(A) RNA-Seq libraries were constructed using the NEB Ultra II RNA Library Prep kit. DNA and RNA sequencing was performed by the Scientific Operations core at the WSI on Pacific Biosciences SEQUEL II (HiFi) and Illumina NovaSeq 6000 (RNA-Seq) instruments. Hi-C data were also generated from leaf tissue of daScuMino1 using the Arima2 kit and sequenced on the Illumina NovaSeq 6000 instrument.

### Genome assembly, curation and evaluation

Assembly was carried out with Hifiasm ( Cheng *et al.*, 2021) and haplotypic duplication was identified and removed with purge_dups ( Guan *et al.*, 2020). The assembly was then scaffolded with Hi-C data ( Rao *et al.*, 2014) using YaHS ( Zhou *et al.*, 2023). The assembly was checked for contamination and corrected as described previously ( Howe *et al.*, 2021). Manual curation was performed using HiGlass ( Kerpedjiev *et al.*, 2018) and PretextView ( Harry, 2022). The mitochondrial and plastid genomes were assembled using MBG ( Rautiainen & Marschall, 2021) from PacBio HiFi reads mapping to related genomes. A representative circular sequence was selected for each from the graph based on read coverage.

A Hi-C map for the final assembly was produced using bwa-mem2 ( Vasimuddin *et al.*, 2019) in the Cooler file format ( Abdennur & Mirny, 2020). To assess the assembly metrics, the *k*-mer completeness and QV consensus quality values were calculated in Merqury ( Rhie *et al.*, 2020). This work was done using Nextflow ( Di Tommaso *et al.*, 2017) DSL2 pipelines “sanger-tol/readmapping” ( Surana *et al.*, 2023a) and “sanger-tol/genomenote” ( Surana *et al.*, 2023b). The genome was analysed within the BlobToolKit environment ( Challis *et al.*, 2020) and BUSCO scores ( Manni *et al.*, 2021; Simão *et al.*, 2015) were calculated.

Table 3 contains a list of relevant software tool versions and sources.

**Table 3.** Software tools: versions and sources.

Software tool	Version	Source
BlobToolKit	4.1.7	https://github.com/blobtoolkit/blobtoolkit
BUSCO	5.3.2	https://gitlab.com/ezlab/busco
Hifiasm	0.16.1-r375	https://github.com/chhylp123/hifiasm
HiGlass	1.11.6	https://github.com/higlass/higlass
Merqury	MerquryFK	https://github.com/thegenemyers/MERQURY.FK
MitoHiFi	2	https://github.com/marcelauliano/MitoHiFi
MBG	-	https://github.com/maickrau/MBG
PretextView	0.2	https://github.com/wtsi-hpag/PretextView
purge_dups	1.2.3	https://github.com/dfguan/purge_dups
sanger-tol/genomenote	v1.0	https://github.com/sanger-tol/genomenote
sanger-tol/readmapping	1.1.0	https://github.com/sanger-tol/readmapping/tree/1.1.0
YaHS	yahs-1.2a.2	https://github.com/c-zhou/yahs

### Wellcome Sanger Institute – Legal and Governance

The materials that have contributed to this genome note have been supplied by a Darwin Tree of Life Partner. The submission of materials by a Darwin Tree of Life Partner is subject to the **‘Darwin Tree of Life Project Sampling Code of Practice’**, which can be found in full on the Darwin Tree of Life website here. By agreeing with and signing up to the Sampling Code of Practice, the Darwin Tree of Life Partner agrees they will meet the legal and ethical requirements and standards set out within this document in respect of all samples acquired for, and supplied to, the Darwin Tree of Life Project.

Further, the Wellcome Sanger Institute employs a process whereby due diligence is carried out proportionate to the nature of the materials themselves, and the circumstances under which they have been/are to be collected and provided for use. The purpose of this is to address and mitigate any potential legal and/or ethical implications of receipt and use of the materials as part of the research project, and to ensure that in doing so we align with best practice wherever possible. The overarching areas of consideration are:

•   Ethical review of provenance and sourcing of the material

•   Legality of collection, transfer and use (national and international)

Each transfer of samples is further undertaken according to a Research Collaboration Agreement or Material Transfer Agreement entered into by the Darwin Tree of Life Partner, Genome Research Limited (operating as the Wellcome Sanger Institute), and in some circumstances other Darwin Tree of Life collaborators.

## Data Availability

European Nucleotide Archive: *Scutellaria minor*. Accession number PRJEB57113; https://identifiers.org/ena.embl/PRJEB57113 ( Wellcome Sanger Institute, 2023). The genome sequence is released openly for reuse. The *Scutellaria minor* genome sequencing initiative is part of the Darwin Tree of Life (DToL) project. All raw sequence data and the assembly have been deposited in INSDC databases. The genome will be annotated using available RNA-Seq data and presented through the Ensembl pipeline at the European Bioinformatics Institute. Raw data and assembly accession identifiers are reported in Table 1.

## Author information

Members of the Royal Botanic Gardens Kew Genome Acquisition Lab are listed here: https://doi.org/10.5281/zenodo.4786680.

Members of the Plant Genome Sizing collective are listed here: https://doi.org/10.5281/zenodo.7994306.

Members of the Darwin Tree of Life Barcoding collective are listed here: https://doi.org/10.5281/zenodo.4893703.

Members of the Wellcome Sanger Institute Tree of Life Management, Samples and Laboratory team are listed here: https://doi.org/10.5281/zenodo.10066175.

Members of Wellcome Sanger Institute Scientific Operations: Sequencing Operations are listed here: https://doi.org/10.5281/zenodo.10043364.

Members of the Wellcome Sanger Institute Tree of Life Core Informatics team are listed here: https://doi.org/10.5281/zenodo.10066637.

Members of the Tree of Life Core Informatics collective are listed here: https://doi.org/10.5281/zenodo.5013541.

Members of the Darwin Tree of Life Consortium are listed here: https://doi.org/10.5281/zenodo.4783558.

## References

[ref-1] Abdennur N Mirny LA : Cooler: Scalable storage for Hi-C data and other genomically labeled arrays. *Bioinformatics.* 2020;36(1):311–316. 10.1093/bioinformatics/btz540 31290943 PMC8205516

[ref-2] Bates A Clayton-Lucey I Howard C : Sanger Tree of Life HMW DNA Fragmentation: Diagenode Megaruptor ^®^3 for LI PacBio. *Protocols.Io.* 2023. 10.17504/protocols.io.81wgbxzq3lpk/v1

[ref-3] Challis R Richards E Rajan J : BlobToolKit - Interactive Quality Assessment of Genome Assemblies. *G3 (Bethesda).* 2020;10(4):1361–1374. 10.1534/g3.119.400908 32071071 PMC7144090

[ref-4] Cheng CS Chen J Tan HY : *Scutellaria baicalensis* and Cancer Treatment: Recent Progress and Perspectives in Biomedical and Clinical Studies. *Am J Chin Med.* 2018;46(1):25–54. 10.1142/S0192415X18500027 29316796

[ref-5] Cheng H Concepcion GT Feng X : Haplotype-resolved *de novo* assembly using phased assembly graphs with hifiasm. *Nat Methods.* 2021;18(2):170–175. 10.1038/s41592-020-01056-5 33526886 PMC7961889

[ref-6] Cole MD Anderson JC Blaney WM : Neo-clerodane insect antifeedants from *Scutellaria galericulata.* *Phytochemistry.* 1990;29(6):1793–1796. 10.1016/0031-9422(90)85018-B

[ref-7] de Boer JGD Quiney B Walter PB : Protection against aflatoxin-B _1_-induced liver mutagenesis by *Scutellaria baicalensis*. *Mutat Res.* 2005;578(1–2):15–22. 10.1016/j.mrfmmm.2005.01.016 16202794

[ref-8] Denton A Yatsenko H Jay J : Sanger Tree of Life Wet Laboratory Protocol Collection V.1. *protocols.io.* 2023. 10.17504/protocols.io.8epv5xxy6g1b/v1

[ref-9] Di Tommaso P Chatzou M Floden EW : Nextflow enables reproducible computational workflows. *Nat Biotechnol.* 2017;35(4):316–319. 10.1038/nbt.3820 28398311

[ref-10] do Amaral RJV Bates A Denton A : Sanger Tree of Life RNA Extraction: Automated MagMax™ mirVana. *protocols.io.* 2023. 10.17504/protocols.io.6qpvr36n3vmk/v1

[ref-11] Doležel J Greilhuber J Suda J : Estimation of nuclear DNA content in plants using flow cytometry. *Nat Protoc.* 2007;2(9):2233–2244. 10.1038/nprot.2007.310 17853881

[ref-12] Georgieva Y Katsarova M Gercheva K : HPLC analysis of flavonoids from *Scutellaria altissima*. *Bulgarian Chemical Communications.* 2019;51:119–123. Reference Source

[ref-13] Grzegorczyk-Karolak I Gołab K Gburek J : Inhibition of advanced glycation end-product formation and antioxidant activity by extracts and polyphenols from *Scutellaria alpina* L. and S. *altissima* L. *Molecules.* 2016;21(6):739. 10.3390/molecules21060739 27314314 PMC6273165

[ref-14] Guan D McCarthy SA Wood J : Identifying and removing haplotypic duplication in primary genome assemblies. *Bioinformatics.* 2020;36(9):2896–2898. 10.1093/bioinformatics/btaa025 31971576 PMC7203741

[ref-15] Harry E : PretextView (Paired REad TEXTure Viewer): A desktop application for viewing pretext contact maps. 2022; [Accessed 19 October 2022]. Reference Source

[ref-16] Henniges MC Powell RF Mian S : A taxonomic, genetic and ecological data resource for the vascular plants of Britain and Ireland. *Sci Data.* 2022;9(1):1. 10.1038/s41597-021-01104-5 35013360 PMC8748506

[ref-17] Howe K Chow W Collins J : Significantly improving the quality of genome assemblies through curation. *GigaScience.* Oxford University Press,2021;10(1): giaa153. 10.1093/gigascience/giaa153 33420778 PMC7794651

[ref-18] Jackson B Howard C : Sanger Tree of Life Sample Homogenisation: Cryogenic Bead Beating of Plants with FastPrep-96. *Protocols.Io.* 2023a. 10.17504/protocols.io.rm7vzxk38gx1/v1

[ref-19] Jackson B Howard C : Sanger Tree of Life HMW DNA Extraction: Automated Plant MagAttract v.4. *Protocols.Io.* 2023b. 10.17504/protocols.io.8epv5xrd5g1b/v1

[ref-20] Jay J Yatsenko H Narváez-Gómez JP : Sanger Tree of Life Sample Preparation: Triage and Dissection. *protocols.io.* 2023. 10.17504/protocols.io.x54v9prmqg3e/v1

[ref-21] Kerpedjiev P Abdennur N Lekschas F : HiGlass: web-based visual exploration and analysis of genome interaction maps. *Genome Biol.* 2018;19(1): 125. 10.1186/s13059-018-1486-1 30143029 PMC6109259

[ref-22] Loureiro J Rodriguez E Dolezel J : Two new nuclear isolation buffers for plant DNA flow cytometry: A test with 37 species. *Ann Bot.* 2007;100(4):875–888. 10.1093/aob/mcm152 17684025 PMC2749623

[ref-23] Manni M Berkeley MR Seppey M : BUSCO Update: Novel and Streamlined Workflows along with Broader and Deeper Phylogenetic Coverage for Scoring of Eukaryotic, Prokaryotic, and Viral Genomes. *Mol Biol Evol.* 2021;38(10):4647–4654. 10.1093/molbev/msab199 34320186 PMC8476166

[ref-24] Morton JK : A cytological study of the British Labiatae (excluding *Mentha*). *Watsonia.* 1973;9:239–246. Reference Source

[ref-25] Oatley G Sampaio F Howard C : Sanger Tree of Life Fragmented DNA clean up: Automated SPRI. *protocols.io.* 2023. 10.17504/protocols.io.q26g7p1wkgwz/v1

[ref-26] Pellicer J Powell RF Leitch IJ : The application of flow cytometry for estimating genome size, ploidy level endopolyploidy, and reproductive modes in plants.In: Besse, P. (ed.) New York, NY: *Methods Mol Biol.* 2021;2222:325–361. 10.1007/978-1-0716-0997-2_17 33301101

[ref-27] Ranjbar M Mahmoudi C : Chromosome numbers and biogeography of the genus *Scutellaria* L. (Lamiaceae). *Caryologia.* 2013;66(3):205–214. 10.1080/00087114.2013.821840

[ref-28] Rao SSP Huntley MH Durand NC : A 3D map of the human genome at kilobase resolution reveals principles of chromatin looping. *Cell.* 2014;159(7):1665–1680. 10.1016/j.cell.2014.11.021 25497547 PMC5635824

[ref-29] Rautiainen M Marschall T : MBG: Minimizer-based sparse de Bruijn Graph construction. *Bioinformatics.* 2021;37(16):2476–2478. 10.1093/bioinformatics/btab004 33475133 PMC8521641

[ref-30] Rhie A McCarthy SA Fedrigo O : Towards complete and error-free genome assemblies of all vertebrate species. *Nature.* 2021;592(7856):737–746. 10.1038/s41586-021-03451-0 33911273 PMC8081667

[ref-31] Rhie A Walenz BP Koren S : Merqury: reference-free quality, completeness, and phasing assessment for genome assemblies. *Genome Biol.* 2020;21(1): 245. 10.1186/s13059-020-02134-9 32928274 PMC7488777

[ref-32] Shang X He X He X : The genus *Scutellaria* an ethnopharmacological and phytochemical review. *J Ethnopharmacol.* 2010;128(2):279–313. 10.1016/j.jep.2010.01.006 20064593

[ref-33] Shen J Li P Liu S : Traditional uses, ten-years research progress on phytochemistry and pharmacology, and clinical studies of the genus *Scutellaria.* *J Ethnopharmacol.* 2021;265: 113198. 10.1016/j.jep.2020.113198 32739568

[ref-34] Simão FA Waterhouse RM Ioannidis P : BUSCO: assessing genome assembly and annotation completeness with single-copy orthologs. *Bioinformatics.* 2015;31(19):3210–3212. 10.1093/bioinformatics/btv351 26059717

[ref-35] Stace CA Thompson H Stace M : New flora of the British Isles. 4th Edn. C&M Floristics,2019. Reference Source

[ref-36] Stroh PA Walker KJ Humphrey TA : Plant Atlas 2020. Mapping changes in the distribution of the British and Irish flora.Durham: Botanical Society of Britain and Ireland,2023;2. 10.2307/j.ctv2x6f08m

[ref-37] Surana P Muffato M Qi G : sanger-tol/readmapping: sanger-tol/readmapping v1.1.0 - Hebridean Black (1.1.0). *Zenodo.* 2023a; [Accessed 21 July 2023]. 10.5281/zenodo.7755669

[ref-38] Surana P Muffato M Sadasivan Baby C : sanger-tol/genomenote (v1.0.dev). *Zenodo.* 2023b. 10.5281/zenodo.6785935

[ref-39] Vasimuddin M Misra S Li H : Efficient Architecture-Aware Acceleration of BWA-MEM for Multicore Systems. In: *2019 IEEE International Parallel and Distributed Processing Symposium (IPDPS)*. IEEE,2019;314–324. 10.1109/IPDPS.2019.00041

[ref-41] Wellcome Sanger Institute: The genome sequence of the Lesser Skullcap, *Scutellaria minor* Huds., 1762. European Nucleotide Archive. [dataset], accession number PRJEB57113,2023.

[ref-40] Zhou C McCarthy SA Durbin R : YaHS: yet another Hi-C scaffolding tool. *Bioinformatics.* Edited by Alkan, C.2023;39(1): btac808. 10.1093/bioinformatics/btac808 36525368 PMC9848053

