# Supplementary material for: The genome sequence of the Lesser Skullcap, Scutellaria minor Huds., 1762 (Lamiaceae)
Source: Wellcome Open Res. 2024 Mar 20;9:165. [Version 1] doi: 10.12688/wellcomeopenres.21164.1 (PMC11350328; doi:10.12688/wellcomeopenres.21164.1)
Supplement: Peer review history

# Reviewer report: Reviewer response for version 1

**DOI:** 10.21956/wellcomeopenres.23409.r80050

**Published:** 2024-08-27

**Authors:** Liangsheng Zhang

**Affiliations:** 1 Genomics and Genetic Engineering Laboratory of Ornamental Plants, College of Agriculture and Biotechnology, Zhejiang University, Hangzhou, Zhejiang, China

Genome assembly is a crucial step in the field of genomics, providing a comprehensive view of the genetic information contained within an organism. When the assembly is successful, it lays a solid foundation for further analysis and understanding of the organism's biology. The inclusion of mitochondrial and chloroplast genomes in the assembly is particularly valuable, as these organelles play key roles in cellular energy production and have their own unique genetic systems. While annotations may not be necessary for certain article formats, providing annotations for the genome is highly recommended. These annotations should include details such as protein and coding DNA sequences (CDS), as well as information in the General Feature Format (GFF), which can greatly facilitate the use of the genome by the broader scientific community. Annotations not only make the data more accessible but also pave the way for a simplified version of the genome to be developed. Annotations are crucial for deciphering the functional elements within the genome. They help identify the parts of the DNA that code for proteins and those that have regulatory roles.

Are sufficient details of methods and materials provided to allow replication by others?

Yes

Is the rationale for creating the dataset(s) clearly described?

Yes

Are the datasets clearly presented in a useable and accessible format?

Yes

Are the protocols appropriate and is the work technically sound?

Yes

Reviewer Expertise:

plant genome and bioinformatics

I confirm that I have read this submission and believe that I have an appropriate level of expertise to confirm that it is of an acceptable scientific standard.

# Reviewer report: Reviewer response for version 1

**DOI:** 10.21956/wellcomeopenres.23409.r90033

**Published:** 2024-08-09

**Authors:** Lei Yu

**Affiliations:** 1 College of Agronomy, Yunnan Urban Agricultural Engineering and Technological Research Center, Kunming University, Kunming, Yunnan, China

The authors have presented a study entitled " The genome sequence of the Lesser Skullcap, *Scutellaria minor *Huds., 1762 ( *Lamiaceae*)". The paper is clear, concise and the quality of the figures and panels is excellent. Here, the authors revealed whole genome, mitochondrial and plastid genome of *Scutellaria minor* which will strengthen the understanding of Scutellaria. However, there are some points need to be considered.

1. The genome size of *Scutellaria minor* has been determined previously using flow cytometry (420 Mb) and genome sequencing (341.8 Mb) in previous studies, which shows some variation compared to the findings of the current study. It is important to provide the reasons behind these discrepancies in genome size.
2. The result need to be reorganized, not just show the figure. They must to be fully explained and described.
3. The manuscript lacks information regarding the coverage of the genome assembly. It would be valuable to include details about the sequencing depth and coverage achieved in the study. This information is essential for assessing the reliability and completeness of the genome assembly.
4. Figure 5 lacks chromosome numbering.
5. It has been mentioned that k-mer was used for analyze the integrity of genome assembly, without any mention of the k-mer selection process and the specific value of k-mer. Different kmer values have great influence on QV values and kmer integrity results.

Are sufficient details of methods and materials provided to allow replication by others?

Yes

Is the rationale for creating the dataset(s) clearly described?

Yes

Are the datasets clearly presented in a useable and accessible format?

Yes

Are the protocols appropriate and is the work technically sound?

Yes

Reviewer Expertise:

crop breeding

I confirm that I have read this submission and believe that I have an appropriate level of expertise to confirm that it is of an acceptable scientific standard.
